# Parental childhood growth and offspring birthweight: Pooled analyses from four birth cohorts in low and middle income countries

**DOI:** 10.1002/ajhb.22614

**Published:** 2014-09-03

**Authors:** OY Addo, AD Stein, CHD Fall, DP Gigante, AM Guntupalli, BL Horta, CW Kuzawa, N Lee, SA Norris, C Osmond, P Prabhakaran, LM Richter, HPS Sachdev, R Martorell

**Affiliations:** 1 Hubert Department of Global Health, Rollins School of Public Health, Emory UniversityAtlanta, Georgia; 2 MRC Lifecourse Epidemiology Unit, University of SouthamptonSouthampton, United Kingdom; 3 Postgraduate Program in Epidemiology, Universidade Federal de PelotasPelotas, Brasil; 4 Center for Research on Ageing, University of SouthamptonSouthampton, United Kingdom; 5 Department of Anthropology, Northwestern UniversityEvanston, Illinois; 6 Office of Population Studies Foundation, University of San CarlosCebu, Philippines; 7 MRC/Wits Developmental Pathways for Health Research Unit, Faculty of Health Sciences, University of the Witwatersrand, South Africa and Human Sciences Research CouncilSouth Africa; 8 Public Health Foundation of IndiaNew Delhi, India; 9 Department of Pediatrics, Sitaram Bhartia Institute of Science and ResearchNew Delhi, India; COHORTS – Consortium on Health Orientated Research in Transitional Societies

## Abstract

**Objective:**

Associations between parental and offspring size at birth are well established, but the relative importance of parental growth at different ages as predictors of offspring birthweight is less certain. Here we model parental birthweight and postnatal conditional growth in specific age periods as predictors of offspring birthweight.

**Methods:**

We analyzed data from 3,392 adults participating in four prospective birth cohorts and 5,506 of their offspring.

**Results:**

There was no significant heterogeneity by study site or offspring sex. 1SD increase in maternal birthweight was associated with offspring birthweight increases of 102 g, 1SD in maternal length growth 0–2 year with 46 g, and 1SD in maternal height growth Mid-childhood (MC)-adulthood with 27 g. Maternal relative weight measures were associated with 24 g offspring birth weight increases (2 year- MC) and 49 g for MC-adulthood period but not with earlier relative weight 0–2 year. For fathers, birthweight, and linear/length growth from 0–2 year were associated with increases of 57 and 56 g in offspring birthweight, respectively but not thereafter.

**Conclusions:**

Maternal and paternal birthweight and growth from birth to 2 year each predict offspring birthweight. Maternal growth from MC-adulthood, relative weight from 2-MC and MC-adulthood also predict offspring birthweight. These findings suggest that shared genes and/or adequate nutrition during early life for both parents may confer benefits to the next generation, and highlight the importance of maternal height and weight prior to conception. The stronger matrilineal than patrilineal relationships with offspring birth weight are consistent with the hypothesis that improving the early growth conditions of young females can improve birth outcomes in the next generation. Am. J. Hum. Biol. 27:99–105, 2015. © 2014 The Authors American Journal of Human Biology Published by Wiley Periodicals, Inc.

Poor nutrition in early life may influence fetal and infant growth of future generations via genetic, epigenetic, physiological, and other mechanisms. Improved nutrition prior to age 2 year has been linked to taller adult stature, improved cognitive and school performance and in males, only, increased economic productivity and earnings (Hoddinott et al., [Bibr b12],[Bibr b11]; Martorell et al., 2010; Stein et al., 2010); these characteristics can influence offspring birthweight by improving, among others, household welfare and the condition of women. Nutritional supplementation in Guatemalan girls was associated with substantial increases in their offspring' birthweight and height and head circumference in childhood; supplementation in boys was not associated with offspring' growth (Behrman et al., 2009). The size of the uterus and the ovaries are reduced in women born with intrauterine growth restriction, which can influence reproductive outcomes (Ibaňez et al., 2003). Other mechanisms for intergenerational influences are also possible and are not mutually exclusive. These include shared environments and genes as well as epigenetic changes resulting from nutritional exposures during embryogenesis, intra-uterine and early postnatal life (Burdge et al., 2007; Martorell and Zongrone, 2012; Pembrey et al., 2013).

Past studies have demonstrated associations of parental anthropometry birthweight (and adult height and Body Mass Index, BMI) with offspring birth size, with most but not all finding evidence for stronger matrilineal than patrilineal inheritance of birthweight, BW (Addo et al., 2013; Kuzawa and Eisenberg, 2012; Mattsson and Rylander, 2013; Ramakrishnan et al., 1999; Subramanian et al., 2009; Veena et al., 2004).

Adult height and BMI are cumulative measures and little is known about the relative importance for offspring birthweight of parental growth at specific periods during childhood. Such information would be useful for identifying critical periods during which growth failure has greater consequences and for setting priorities for nutrition interventions. To our knowledge only one study, from Pelotas, Brazil, has related growth during childhood in mother and fathers to offspring birthweight (Horta et al., 2009). The authors reported that mothers who gained weight faster than the population mean from birth to 20 months, but not from 20 months to 42 months, had heavier firstborn babies. In contrast, paternal weight gain at these ages was unrelated to offspring BW.

Here we build upon past work in this area by including three cohorts in addition to the Pelotas cohort and by examining all births, not just first-borns, while also including measures of growth in height and weight from birth through adulthood. Specifically, we assess the relative importance of parental birthweight and growth during different growth intervals from birth to adulthood as predictors of offspring birthweight in four cohorts from low and middle income countries (LMICs).

## Study methods

The data for this study come from 4 of the 5 birth cohorts that participate in the Consortium on Health Orientated Research in Transitional Societies (COHORTS) (Richter et al., 2012). We use data from the 1982 Pelotas Birth Cohort–Brazil (Victora and Barros, 2006), the Institute of Nutrition of Central America and Panama Nutrition Trial Cohort–Guatemala (Stein et al., 2008), the New Delhi Birth Cohort–India (Sachdev et al., 2005) and the Cebu Longitudinal Health and Nutrition Survey CLHNS–Philippines) (Adair et al., 2011). The Birth to Twenty Cohort (BT20)–South Africa (Richter et al., 2007), also a member of COHORTS, is the youngest, with only a small numbers of births to date and was not included in this analysis. Attrition information by site for the last follow-up is provided elsewhere (Richter et al. 2012). The number of people not traced into adulthood varied between 1% in the Philippines and 17% in Brazil. Other causes of attrition were death, usually in early childhood, and migration out of the area or country. The percentage of the original cohort members examined at the last follow-up was over 65% in three cohorts (Brazil, Guatemala, and the Philippines) and 29% in India, where nearly half the original participants moved out of the study area and could not be examined at follow-up. Prior analyses of COHORTS data indicate that estimated coefficients do not seem to have been biased by attrition or missing data (Adair et al., 2013). Finally, between 41 and 83% of adults examined at last follow-up (with information on birthweight and height and weight at other periods including adulthood) provided birthweight information for at least one child.

## Data collection

### Parental measures

Parental birth weights and postnatal anthropometry were measured following site-specific protocols by trained observers. Birthweight was measured in hospitals at delivery in Brazil, in the community within 72 h of birth in India, in hospitals or at home by birth attendants in The Philippines and by a project nurse at home or in a health post in Guatemala. Mid childhood (MC) heights and weights were measured at variable ages due to site-specific differences in data collection schedules. It was at 4 years in Brazil, Guatemala and India, and 8.5 years in the Philippines. We used *growth* variables of each parent as study exposures. We defined maternal and paternal childhood stunting using the WHO height-for-age z scores (HAZ) ≤2SD cut-off at 2 year (WHO Multicentre Growth Reference Study Group, 2006).

### Offspring measures

Offspring birth weights were reported by the parent, except in Guatemala where they were obtained by measurement for births before 1999 and by recall later (Behrman et al., 2009; Ramakrishnan et al., 1999). Offspring birthweight and low birthweight (LBW, <2,500 g) were considered as study outcomes.

## Other variables

We included several variables as covariates: parental early childhood household socioeconomic status (quintiles, lowest as reference category), parental birth order (categorized into firstborns and non-firstborns), maternal age at delivery of offspring (categorized as <18y or ≥18y), type of nutrition supplementation received in Guatemala, and offspring sex. Parental early childhood SES was represented by father's income in Brazil, father's occupation and mother's education in India, and by asset scores, derived from principal component analyses (PCA) of household items for all other sites. As the parents in the Guatemala cohort had participated as children in a nutrition supplementation trial that improved linear growth (Habicht et al., 1995), we created a three category variable (one for each of the Guatemala 2 trial arms, and the 3rd category for the other sites that had no intervention). In pooled mixed models we also controlled for study site.

## Data management

### Analytic sample and inclusion criteria

The primary inclusion criterion for the analytic study sample was availability of birthweight for the parent and the child (*n* = 4,906). Hundred and seven (1.9%) pairs were excluded because the child was not a singleton.

### Parental conditional growth

We used *conditional height* and *conditional relative weight* variables to assess growth (length/height and weight) from birth through adulthood (Adair et al., 2013; Keijzer-Veen et al., 2005; Li et al. 2003). The conditional variables are residuals from site- and sex-specific linear regression models. Conditional height is present length or height accounting for previous length or height, and weight (but not present weight). Conditional relative weight is present weight accounting for present height and all previous weight and height measures. For example, adult conditional relative weight is derived from a regression of adult weight on adult height, weight and height at mid-childhood, weight and length when aged 2 years, and birthweight. The conditionals may be interpreted as the deviation from the child's own predicted growth trajectory in relation to that of its peers, and hence measure relative growth within an age interval (Adair et al., 2013).Conditional growth variables were expressed in SD units to allow for comparisons of coefficients across measures, periods and parental sex. As conditional height and relative weight variables are by definition uncorrelated, they can be included in a single multivariate model without causing variance inflation. The conditional variables were estimated for the periods: birth-2 year, 2 year-Mid-childhood (MC) and MC-adulthood. For convenience, we name these periods *early*, *middle*, and *late* childhood periods of maternal growth. Because birth length was not available for Brazil, conditional variable derivations used birthweight as a measure of birth size for all sites. Conditional height refers to growth in length/height while conditional relative weight refers to weight gain independent of linear growth.

## Statistical methods

Descriptive characteristics are presented as means (SD) for continuous variables and as percentages for categorical variables. We estimated associations of parental birthweight and conditional heights and relative weights with offspring birthweight, separately for mothers and fathers, using mixed linear models adjusted for parental level covariates, offspring sex and study site as fixed effects. As we found significant intra family clustering of offspring birthweights (intra cluster correlations (ICC) = 28.7–40.5%, *P* < 0.0001), we used mixed regression modelling to nest births within each parent as random intercepts. The classical sandwich estimator (Diggle et al., 2002b) in variance estimation for fixed-effects was applied. Heterogeneity was tested using site-sex interaction term as a fixed effect. No statistical heterogeneity (*P*_interaction_ > 0.05) was found for any of the models. We therefore carried out pooled analysis with adjustment for site and offspring sex as fixed effects.

We examined the associations of parental birthweight and childhood conditional linear growth with offspring LBW status. We used generalized linear mixed models (GLMM) to estimate the prevalence ratios (PR) of LBW adjusted for random effects with parental childhood conditional growth variables as the chief model predictors. Similarly, we related parental childhood stunting with offspring LBW status in a separate mixed logit model. Since prevalence estimates of LBW and parental childhood stunting were not low (13–84%), PR (95% CI) with robust variance (Zou, 2004) were considered to be more precise measures of association and were used instead of odds ratios.

## Sensitivity analyses

All parental conditional variable derivation were based on birthweight rather than birthlength (unavailable for Brazil). The results of a sensitivity analysis showed very similar estimates using either birthlength or birthweight for the 3 cohorts that had both length and birthweight. Also, sensitivity analyses indicated no significant differences in results using growth measures obtained at 8.5 year (Philippines) vs. those obtained at 4 year (other 3 sites), as previously reported (Adair et al., 2013).

Offspring gestational age was only available for Guatemala and the Philippines and as a categorical variable (preterm: <37, term: 37–41 and post-term: >41 weeks) in India and was not included in the analysis. We conducted sensitivity analyses by first converting continuous gestational ages (GA) in Guatemala and the Philippines to categorical variables in order to carry out a pooled analysis with India. The results of this pooled analysis involving the three sites with GA were very similar to the main analyses with all four sites but not controlled for gestational age.

## Missing data

About 22% of parents were missing one or more measure of childhood growth and SES. We used multiple imputation methods under an assumption of missingness at random and generated 15 datasets with imputed values (Little and Rubin, 2002) using the Amelia II package (Honaker et al., 2009). Imputation accuracy was assessed with over-impute diagnostics graph confidence bands and was found to be adequate (Honaker et al., 2009). Combined inference estimates were obtained from the 15 imputation datasets using Rubin's Imputation inference rules. Sensitivity analyses conducted using mother-offspring or father-offspring pairs with available data (list-wise deletion) were identical to those obtained from imputation analyses, albeit with wider confidence intervals (results not shown). We therefore present results from the imputation analyses. Two-tailed *P*-values <0.05 were considered statistically significant. All analyses were conducted with PC-SAS version 9.3 (SAS Institute, Cary, NC), and R (R Core Team, 2014) software with the package ‘lme4'.

## RESULTS

The ages of the parents at the last follow-up are shown in Table1. Mean maternal and paternal adult heights were 156.6 cm and 164.4 cm, respectively. Prevalence of childhood stunting at 2 year ranged from 22 to 84% and was similar in fathers and mothers (Table2). Low birthweight prevalence ranged between 11.5 and 20.0% across all sites for offspring of mothers and fathers. The mean number of births-to-date ranged from 1.5 for India to 2.2 in Guatemala. Fathers of the Brazilian and Indian cohorts reported fewer children in comparison to those from Guatemala and the Philippines.

**Table 1:** Characteristics of the participating cohorts

Cohort	Design	Enrollment year	Year and age of cohort members at most recent follow-up	Cohort description
Pelotas birth cohort, Brazil	Prospective cohort	1982	2012, 30 years	Children born in the city's maternity hospital (>99% of all births in 1982). All social classes included.
INTCS, Guatemala	Community Trial	1969–1977	2004, 26 −41 years	Intervention trial of a high-energy and protein supplement in women, and children <7 years in 1969 and born during 1969–1977 in four villages.
New Delhi Birth Cohort, India	Prospective Cohort	1969–1972	1998–2002, 26–32 years	Babies born to an identified population of married women living in a defined area of Delhi. Primarily middle class sample
CLHNS, Cebu Philippines	Prospective Cohort	1983–1984	2009, 25 years	Pregnant women living in 33 randomly selected neighborhoods; 75% urban. All social classes included.

**Table 2:** Characteristics of parents and their offspring in four birth cohorts (3,392 parents; 5,506 offspring)

	Pooled results mean (SD)/range	Brazil mean (SD) /range	Guatemala mean (SD)/range	India mean (SD) /range	Philippines mean (SD) /range
Mothers (*N* = 2,031)		***n*= 1063**	**(*n* = 179)**	**(*n* = 285)**	**(*n* = 504)**
Firstborn status (%)	19.0	34.9	11.4	9.2	20.3
Birthweight (kg)	3.0 (0.5)	3.1 (0.5)	2.9 (0.4)	2.8 (0.5)	3.0 (0.4)
Stunted at 2 year (≤2SD) (%)	56.2	23.3	83.9	44.8	72.7
Adult height (cm)	156.6	160.0 (6.0)	150.5 (5.1)	155.3 (5.4)	150.4 (5.5)
Children per mother	1.5 (1–7)	1.6 (1–6)	2.2 (1–7)	1.5 (1–5)	1.7 (1–7)
Offspring of mothers	*N* = 3455	***n* = 1742**	***n* = 317**	***n* = 478**	***n* = 918**
Male (%)	54.0	51.1	53.7	56.0	55.0
Firstborn status (%)	52.7	61.0	37.5	58.2	54.2
Birth weight (kg)	3.0 (0.5)	3.2 (0.6)	3.0 (0.5)	2.8 (0.5)	3.0 (0.6)
Birth weight <2500 g (%)	16.1	11.5	15.8	17.0	20.0
Fathers	*N* = 1361	***n* = 596**	***n* = 96**	***n* = 342**	***n* = 327**
Firstborn status (%)	21.2	41.0	10.5	11.7	21.7
Birth weight (kg)	3.1 (0.5)	3.3 (0.5)	3.0 (0.4)	2.9 (0.4)	3.0 (0.5)
Stunted at 2 year (≤2SD) (%)	48.0	13.6	65.1	43.3	69.4
Adult height (cm)	164.4 (8.7)	173.5 (6.6)	151.3 (4.5)	169.9 (6.1)	162.5 (5.2)
Children per father	1.5 (1–7)	1.3 (1–4)	2.2 (1–7)	1.4 (1–4)	2.0 (1–6)
Offspring of fathers	*N* =2,051	***n* = 795**	***n* =172**	***n* = 564**	***n* =520**
Male (%)	53.6	50.8	52.9	58.5	52.1
Firstborn status (%)	48.5	60.1	39.5	59.2	35.0
Birth weight (Kg)	3.0 (0.6)	3.3 (0.7)	2.9 (0.5)	2.8 (0.5)	3.0 (0.6)
Birth weight <2500 g (%)	14.7	11.3	16.3	15.3	16.0

Results of mixed linear models describing the association between parental growth in SD units and offspring birthweight are shown in Table3. Also shown are the equivalents, in cm or g, corresponding to one SD unit of linear or relative weight, respectively. For mothers, 1 SD of conditional linear growth corresponded to 2.7 cm (birth–2 year), 3.5 cm (2–MC), and 5.4 cm (MC-adulthood) in absolute terms and for the fathers they were 4.0 cm (birth–2 year), 3.7 cm (2–MC) and 10.0 cm (MC–adulthood). Similarly, the absolute equivalents in grams per 1 SD of maternal birthweight was 478 g and that for her conditional relative weight gains were 847 g (birth-2 year), 1,072 g (2–MC), and 10,615g (MC–adulthood). Those of the fathers were 510 g/1SD of birthweight, and 921 g (birth–2 year), 1,182 g (2–MC), 10,524 g (MC-adulthood) per 1SD absolute weight gain. Absolute weight gains were higher (>10 kg) for the late childhood period (MC-Adulthood) and similar in both parents.

**Table 3:** Associations between parental height and relative weight gain in specific periods of childhood and offspring birthweight in four birth cohorts (3,392 parents; 5,506 offspring)

Parental growth measures	Offspring birthweight (g)					
	Mothers (*N* = 2,031) and their offspring (*N* = 3,455)			Fathers (*N* =1,361) and their offspring (*N* =2,051)		
	Equivalent per SD of conditional growth	Coefficient (95% CI)	*P-*value	Equivalent per SD of conditional growth	Coefficient (95% CI)	*P-*value
Birthweight (z)	478 g	102.3 (79.5, 125.2)	<0.01	510 g	57.3 (25.9, 88.6)	<0.01
Conditional height 0–2 year (z)	2. 7 cm	45.5 (20.8, 70.3)	<0.01	4.0 cm	55.5 (20.8, 90.3)	<0.01
Conditional height 2 year-MC (z)	3.5 cm	12.4 (−10.6, 35.5)	0.29	3.7 cm	19.9 (−7.6, 47.4)	0.16
Conditional height MC-adulthood(z)	5.4 cm	27.3 (3.5, 51.1)	0.03	10.0 cm	13.9 (−17.6, 45.4)	0. 39
Conditional relative weight gain 0–2 year (z)	847 g	16.6 (−6.7, 39.8)	0.16	921 g	25.6 (−4.7, 55.9)	0.10
Conditional relative weight gain 2 year-MC (z)	1,072 g	24.3 (1.5, 47.0)	0.04	1,182 g	−1.5 (−34.7, 31.6)	0.93
Conditional relative weight gain MC-adulthood(z)	10,615 g	49.1 (25.3, 72.8)	<0.01	10,524 g	8.1 (−21.8, 38.1)	0.60

MC: Mid-childhood is 4 years for Brazil, Guatemala, and India and 8.5 y for Philippines. Models are adjusted for parental early childhood SES (quintiles), maternal/paternal firstborn status, offspring sex and firstborn status, nutrition supplementation status, site(4 categories), maternal age at delivery (<18, ≥18 year), and sibling clustering (with random intercepts). Estimates obtained from multiple imputation analyses of 15 replicate datasets.

In adjusted models, maternal birthweight and postnatal conditional linear/length growth were associated with offspring birthweight. The increases per one SD were 102.3 g (95% CI: 79.5–125.2) with maternal birthweight, 45.5 g (95% CI: 20.8–70.3) with length growth 0–2 year, and 27.3 g (95% C: 3.5–51.1) with height growth MC-Adulthood.

Paternal birthweight and conditional linear/length growth 0–2 year were associated with 57.3 g (95% C: 25.9–88.6), and 55.5 g (95% C: 20.8–90.3) increases in offspring birthweight, respectively; there were no associations with 2 year-MC or MC-adulthood. There were significant associations between maternal relative weight for 2 year-MC (β = 24.3 g, 95% CI: 1.3–47.0) and MC – adulthood (β = 49.1 g, 95% CI: 25.3–72.8) and offspring birthweight. There were no significant associations with offspring birthweight for any of the paternal relative weight measures (Table3).

Associations between parental conditional childhood growth and risk (prevalence ratio) of offspring LBW status are shown in Table4. Parental birthweight and linear growth from 0 to 2 year were independently associated with reduced risk of offspring low birthweight, with prevalence ratio (PR) = 0.7 (95% CI: 0.6–0.8) and PR = 0.8 (95% CI: 0.7–0.9), respectively for mother-offspring; and (PR) = 0.87 (95% CI: 0.8–1.0) and PR = 0.80 (95% CI: 0.7-0.9), respectively for father-offspring models. Associations were nonsignificant for the middle and late periods of paternal linear growth. With respect to relative weight gain, there was a significant association with risk of LBW only for maternal conditional relative weight gain from 2 year–MC (PR = 0.9 (95% CI: 0.8 – 1.0).

**Table 4:** Associations between parental height and relative weight gain in specific periods of childhood and offspring low birthweight in 4 birth cohorts (3,392 parents; 5,506 offspring)

Paternal growth measures	Prevalence ratio, PR (95% CI) for low birthweight (<2,500 g)			
	Mothers (*N* = 2,031) and their offspring (*N* = 3,455)		Fathers (*N* = 1361) and their offspring (*N* = 2,051)	
	PR (95% CI)	*P*-value	PR (95% CI)	*P-*value
Birthweight (z)	0.69 (0.62, 0.77)	<0.01	0.87 (0.77, 0.99)	0.05
Conditional height 0–2 year (z)	0.84 (0.74, 0.94)	<0.01	0.80 (0.69, 0.94)	<0.01
Conditional height 2-MC (z)	0.97 (0.87, 1.09)	0.61	0.95 (0.83, 1.08)	0.41
Conditional height MC-adulthood (z)	0.96 (0.86, 1.08)	0.51	1.00 (0.87, 1.15)	0.99
Conditional relative weight gain 0–2 year (z)	1.01 (0.90, 1.13)	0.87	0.93 (0.80, 1.1)	0.29
Conditional relative weight gain 2 year-MC (z)	0.89 (0. 80, 0.99)	0.05	1.04 (0.89, 1.21)	0.60
Conditional relative weight gain MC-adulthood (z)	0.93 (0.84, 1.03)	0.18	0.99(0.86, 1.13)	0.86

MC: Mid-childhood, denoted by 4 years for Brazil, Guatemala, India and 8 years for Philippines. Models are also adjusted for parental early childhood SES (quintiles), maternal/paternal firstborn status, offspring sex and firstborn status, nutrition supplementation status, site (4 categories), maternal age at delivery (<18, ≥18 year and sibling clustering (with random intercepts).PR for **t**eenage mother delivery (<18 year vs. ≥18 year) for LBW is 1.47 (95% CI: 1.10, 2.00, *P*=0.012). Estimates obtained from multiple imputation analyses of 15 replicate datasets.

We also related parental childhood stunting to offspring birthweight and risk of LBW **(**Table5). Maternal stunting at 2 year (compared to nonstunted) was associated with a 1.6-fold increase (PR = 1. 6, 95% CI: 1.25–2.05) in the risk for offspring LBW and with a 107.6 g (95% CI:−160.3 to −54.8) decrease in offspring birthweight. Paternal childhood stunting was not significantly associated with offspring birthweight or LBW risk.

**Table 5:** Associations between parental stunting at 2 year and offspring birth weight and offspring low birthweight status in 4 birth cohorts (3,392 parents; 5,506 offspring)

Parental stunting status	Birth weight, grams (mixed linear model)		Low birth weight (mixed logit model)	
	Coefficient (95% CI)	*P-*value	Prevalence ratio (95% CI)	*P-*value
Maternal stunting at 2 years	−107.6 (−160.3, −54.8)	<0.01	1.60 (1.25, 2.05)	<0.01
Paternal stunting at 2 year	−28.7 (−95.3, 37.9)	0.40	1.17 (0.86, 1.59)	0.31

Mixed linear and logistic models are adjusted for parental early childhood SES (quintiles), maternal/paternal firstborn status, offspring sex and firstborn status, nutrition supplementation status, site (4 categories), maternal age at delivery (<18, ≥18 year), and sibling clustering (with random intercepts). Estimates obtained from multiple imputation analyses of 15 replicate datasets.

## DISCUSSION

We find that parental birthweight and linear growth from birth to 2 year, but not later, are associated with offspring birthweight. Paternal relative weight gain, independent of linear growth, was not associated with offspring birthweight but maternal relative weight gain was significant for 2 year-MC and MC-adult, respectively. Finally, parental stunting at 2 year, a summary indicator of growth failure in early life, was associated with reductions in birthweight and was predictive of higher risk of low birth weight.

Associations between parental and offspring birthweight are well-known, with most such studies reporting a stronger relationship for maternal birthweight than for paternal birth weight (Kuzawa and Eisenberg, 2012; Mattsson and Rylander, 2013; Ramakrishnan et al., 1999). What is novel in our analysis is that linear growth from 0 to 2 year is strongly associated with offspring birthweight and that the strength of this relationship is similar for both mothers and fathers. Our results contradict a prior study from Brazil (Horta et al., 2009), which found a relationship between weight gain from 0 to 20 months in mothers but not fathers (Horta et al., 2009) (for firstborns only). In the present study, the specific findings for Brazil, with a much larger sample, 1,659 parents and 2,537 offspring (included all births and not firstborns only), are statistically significant for linear growth from 0 to 2 year for both fathers and mothers (results not shown).

The fact that the coefficients for relationships between linear growth (0–2 year, 2 year-MC and MC-adulthood; Table3) and offspring birthweight are very similar for mothers and fathers indicates that, it is unlikely that the mechanisms operate solely through the maternal fetal environment. Since we use conditional variables that control for prior measures, we can exclude the influence of parental birthweight *per se* on parental postnatal growth as a pathway. However, the larger coefficient for maternal compared to paternal birthweight does suggest an influence of the maternal fetal environment on offspring birthweight but the fact that the smaller coefficient for paternal birth weight is nonetheless significant suggests that additional, nonmutually exclusive factors are at play, such as genetic, epigenetic, or environmental factors.

Associations between parental linear growth after age 2 year and offspring birthweight were of lower magnitude than for parental birthweight and linear growth from 0 to 2 year. Early growth failure leading to stunting is a marker of systemic dysfunction caused by societal, household, and individual factors that ultimately lead to poor diets and infection. Linear growth failure occurs during a sensitive phase of child development, from conception to 2 year, and co-occurs with poor growth and development in other organ systems. Hoddinott et al. (2013) reported that stunting at 2 year in both men and women from Guatemala was associated with less schooling and lower test scores for reading and intelligence as compared to nonstunted peers. Stunted adults married, on average, spouses with shorter stature and lower grade attainment as compared to nonstunted adults. Women who were stunted typically had their first child at a younger age and had more pregnancies and more children. Stunting also was strongly associated with individuals being more likely to live in poor households as adults. Conversely, better growth, expressed as height *Z*-scores at 2 year, was consistently associated with better adult outcomes. The long-term consequences of early life growth failure are profound and could provide an explanation for our findings. Better linear growth in early life provides pathways through which offspring birthweight may be influenced and examples are as follows: more household parental resources to obtain a better diet and to access health care, better educated parents with better child rearing practices and health seeking behaviors, and a better physiological maternal capacity to nourish and deliver a healthier offspring (which would explain the larger coefficients for maternal birthweight).

The pattern of relationships with conditional relative weight gain differs from that for linear growth. Father's conditional relative weight gain was not related with offspring birthweight and in mothers, coefficients are larger for later compared to earlier changes. Of note is the importance of relative weight gain from MC to adulthood, which reflects acquisition of both lean and fat mass independent of height and thus larger offspring birthweight (a coefficient of 49 g/1 SD change). This potential benefit, however, has to be balanced with the increased risks of maternal obesity and chronic diseases that are associated with relative weight at this period (Adair et al., 2013). Our data came from low and middle income countries and the degree to which they apply to industrialized societies, which have much less growth failure, needs to be assessed especially using conditional growth modeling for assessing relative importance of parental childhood periods of growth. Despite marked variations in early life growth failure across the four sites, associations were similar across sites; this suggests that our findings may indeed apply to many settings.

The use of uncorrelated measures of growth for four lifecourse periods is a strength of this study that allowed us to assess the relative importance of parental growth during specific developmental periods. The use of conditional height disentangled from weight gain allowed us to isolate the role of linear growth from weight gain. The inclusion of four cohorts that provide a sample size of 3,392 parents and 5,506 offspring afforded us statistical power to test observed associations. Another strength of this study is the use of mixed regression (mixed linear and logit) models that controlled for any potential intra family correlations in birthweight (Diggle et al., 2002a; Fritzmaurice et al., 2004).

Several limitations of this study warrant discussion. One limitation of our study is that offspring birthweight was obtained largely by recall. This may have contributed to loss of precision and underestimation of the associations. Birth length data were unavailable for Brazil and as a result we used conditional growth measures conditioned on birthweight and all prior heights for all the four study sites instead. Gestational age (GA) was unavailable for Brazil. Sensitivity analyses indicated similar results in pooled models with categorized GA (for Guatemala, India and Philippines) vs. all four sites pooled without adjustment for GA. Even though we adjusted for several confounding factors, residual confounding still remains a possibility. Finally, since the parental cohorts married mostly spouses from outside the cohorts, we had few couples who were both cohort members. This precluded many types of analyses that would have been useful in teasing apart paternal and maternal influences.

## CONCLUSIONS

Parental birth weight and linear growth from birth to 2 years, but not later, and maternal conditional relative weight (but not paternal) from 2 year through adulthood, are associated with offspring birth weight These findings suggest the importance of adequate nutrition during pregnancy and the first 2 year of life for both parents as well as the role of preconception body composition in mothers. Other mechanisms, such as shared genes, environment, and epigenetic changes, may also contribute to associations between parental growth and offspring birthweight.
